# Pollution and health risk assessment of trace metal in vegetable field soils in the Eastern Nile Delta, Egypt

**DOI:** 10.1007/s10661-022-10199-1

**Published:** 2022-06-29

**Authors:** Ehab A. Ibrahim, El-Metwally M. Selim

**Affiliations:** 1 Cross Pollinated Vegetable Crops Research Department, Horticulture Research Institute, Agricultural Research Center, 9 Cairo University St., Orman, Giza, Egypt; 2 Department of Soil Sciences, Faculty of Agriculture, Damietta University, Damietta, 34517 Egypt

**Keywords:** Carcinogenic risk, Heavy metals, Monitoring, Non-carcinogenic risk, Soil pollution

## Abstract

The accumulation of trace metals in vegetable field soils is of increasing worry because of the potential health hazards and their detrimental effects on soil ecosystems. To investigate the state of trace metal pollution in vegetable field soils, 60 surface soil samples were collected from vegetable fields across the Eastern Nile Delta region, Egypt. The results concluded that the concentrations of Cu, Mn, and Ni were lesser than their corresponding background values, while the concentrations of Cd, Co, Pb, and Zn were exceeding their background values. The pollution indices showed that the studied soil experienced low to moderate contamination and the Cd and Cr contamination was serious. The hazard index values of nine trace metals signified that there was no adverse non-carcinogenic risk for adults and children. The carcinogenic risk of Cd, Co, Ni, and Pb for both age groups was within acceptable limits, while Cr had critical carcinogenic hazards for children. Overall, the quality of studied soils is relatively safe, although some samples impose serious pollution problems of Cd and Cr. Thus, properly monitored trace metals and soil management action should be applied to reduce further soil pollution in vegetable fields in the Eastern Nile Delta.

**Supplementary Information:**

The online version contains supplementary material available at 10.1007/s10661-022-10199-1.

## Introduction

Over the last few decades, agricultural soil pollution by trace metals has received considerable attention because of its toxicity at low content, bioaccumulation, and persistence (Keshavarzi & Kumar, 2020; Orellana et al., 2020). The accumulation of trace metals in agricultural soil can threaten the environmental ecosystem safety such as the decline in soil and groundwater quality (Bassouny & Abbas, 2020). Moreover, it can threaten food security by adverse effects on crop quality and the health of animals. These might cause potential risks to human health through the food supply chain and groundwater consumption (Mo et al., 2020; Salman et al., 2019). The trace metals of agricultural soil can mainly enter the human body through three exposure routes: ingestion, inhalation, and skin contact (Castro-González et al., 2017). The migration and accumulation of trace metals in agricultural soils are associated with both the chemical and physical properties of soil (Huang et al., 2020). Agricultural soil is classified as polluted when the trace metals concentration in its bulk horizons is higher than the baseline values taken as upper limits for non-contaminated soils (Santos-Francés et al., 2017).

Generally, the trace metals in agricultural soil come from natural processes and/or anthropogenic activities (Orellana et al., 2020). The natural origin of trace metals is related to soil parent materials (Yu et al., 2021)*.* The contribution of anthropogenic activities to the trace metals in rural soil was a lot more than the natural source (Ali et al., 2019). Agricultural activities, especially the utilization of organic and inorganic fertilizer, the use of large quantities of pesticides, and the use of low-quality water are the major sources of trace metals caused by anthropogenic activities (Yang et al., 2018). The large input of agrochemicals leads to soil degradation and pollution, especially in vegetable fields (Jacob and Kakulu, 2012; Singh et al., 2012). Soil pollution poses an important threat to food security by both decreasing crop yields because of the toxicity of pollutants and by causing unsafe crop yields for animals and humans.

Trace metals such as Co, Cu, Cr, Mn, Ni, and Zn are important elements that must be present in trace amounts for plant growth and development (Kuerban et al., 2020). However, when contaminations exceed natural levels in the soil, not only is soil degradation occurring but crop yields and human health can also be affected (Ali et al., 2019). Thus, it is essential to estimate the potential health risk associated with trace metal pollution in agricultural soil. Moreover, as well as imperiling human health and the environment, soil contamination can likewise lead to serious economic losses (Kucher et al., 2015).

The Nile Delta of Egypt covers a large area of agricultural land and represents a vital economic sector. The continuous urbanization and industrialization of the Nile Delta and its surroundings have prompted a rise in the pollution of water resources and soils, causing a probable health hazard (Mohamed, 2017). The rapid increase in soil contamination in Egypt has turned into a real danger to people’s health and the economy. Egyptian farmers had shifted the use of their land for crop production to several seasons per year. Thus, they use excessive amounts of mineral fertilizers and pesticides without any guidelines to increase crop productivity and reduce crop losses, especially in the Nile Delta (Hashim et al., 2017). Moreover, domestic wastewater is reused for agricultural irrigation in rural areas in this region (El Gohary, 2015). These practices have been causing the aggregation of trace metals in soil and the degradation of agrarian soil (Omran, 2016; Salman et al., 2017; Abou El-Anwar et al., 2018; Itta et al., 2019).

Several studies have been carried out over the past few years on trace metal pollution of agrarian soil and the ecological risk to soils in different regions in Egypt. The Nile water receives a lot of pollutants from wastewater discharging through agricultural drains and industrial sources. Thus, the trace metals pollution transferred from the water to the soil has accumulated with time (Abou El-Anwar, 2018, 2019; Khalifa & Gad, 2018; Darwish & Pöllmann, 2015). Sometimes, farmers depend on drainage water for irrigating their soils in such an area (Itta et al., 2019; Abu Khatita et al., 2020). These indicate that agricultural soil pollution might be influenced by human activities and requires more attention in the future.

Pollution indices have been widely and efficiently applied to provide a comprehensive description of the status of trace metal contamination in soil. Shokr et al. (2016) found that the pollution load index was > 1 in most soil samples from the middle part of the Nile Delta, showing considerable to high polluted classes with trace metals. Omran (2016) found that pollution indices revealed that the soil of the Bahr El Baqar area falls under moderate to a very high load of trace metals. Khalifa and Gad (2018) found the highest degrees of pollution and potential ecological risk in the soil of the Quessna district in the Southwestern Nile Delta. Salman et al. (2019) found that the pollution indices revealed that the soil samples from Orabi farms, El Obour city were fluctuating from considerable pollution, as per the potential ecological risk index values, to a high polluted pattern as per the pollution load index and pollution degree results. Abou El-Anwar et al. (2018) found that the pollution indices showed that the agricultural soil at Aswan and Luxor can be considered generally moderately polluted with heavy metals, but they have a very high ecological risk with Cd. However, studies on the heavy metal pollution of agricultural soil and the ecological risk, particularly in the Eastern Nile Delta ecosystems, remain limited.

The pollution characteristics of trace metals in agrarian soil might vary with various types of crops, particularly when their production continues for many years (Yu et al., 2021). The agrochemicals are applied at higher rates in vegetable crops than in most others. Excessive amassing of trace metals in vegetable field soil through the long-term application of agrochemicals and by other sources may result in soil pollution (Islam et al., 2018). Trace metals accumulate at high levels in the vegetable edible parts when contrasted with fruit or grain crops. The consumption of vegetables cultivated in soil that is contaminated with trace metals can cause clinical problems to human health (El Gohary, 2015).

In Egypt, the studies on trace metal pollution of soil in vegetable fields are very limited on large or moderate regional scales. Moreover, the studies on associated human health risks are not common. Further studies for human health risk evaluation are needed to explore potential risks for humans from the pollution of trace metals in agrarian soil in the Eastern Nile Delta area (Khalifa & Gad, 2018). Thus, monitoring the status of trace metal pollution in the soil of vegetable areas is necessary for estimating the potential health risk and adequate management of these pollutants as well as promoting food safety. Thus, the three main objectives of this study were (1) to determine the concentrations, sources and spatial distributions of trace metals in the vegetable field soil of the Eastern Nile Delta area, (2) to assess the trace metal pollution of vegetable field soil, and (3) to evaluate the potential health risk of the trace metals based on various exposure routes.

## Materials and methods

### Study area

**Fig. 1:**
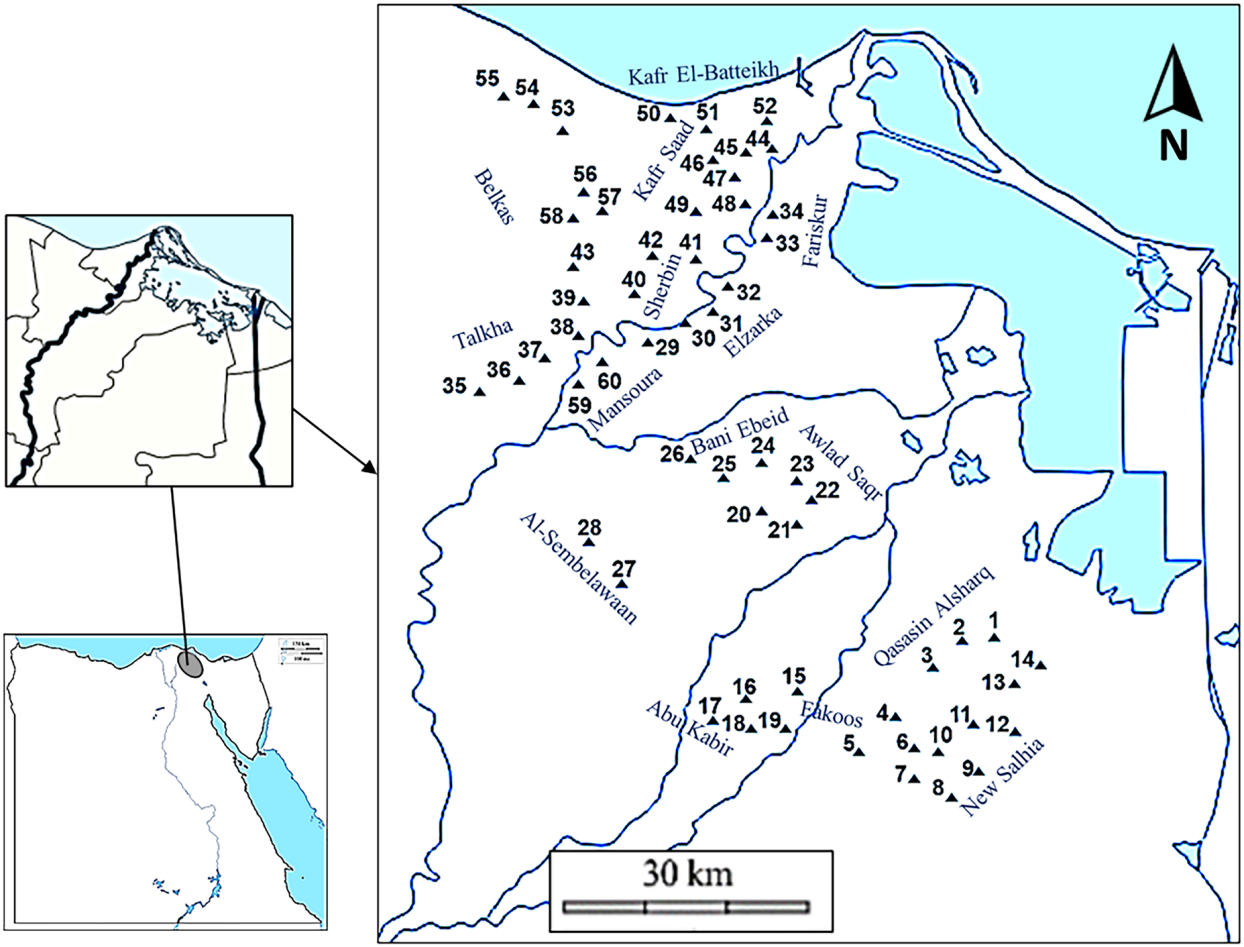
Map of the study area and sampling sites of trace metals in vegetable field soils in the Eastern Nile Delta

The study area is located between latitudes 30° 35′ and 31° 29′ N and longitudes 31° 18′ and 32° 04′ E (Fig. 1). It covers a major part of the eastern portion of the Nile Delta, including Damietta, Dakahlyia, and El-Sharkia Governorates. It has a hot arid summer and low rainy winter, with the total yearly precipitation around 167 mm/year falls mainly between October and March. The average annual temperature ranges are 24–35 °C in summer and 8–20 °C in winter. The soil types are varied from clay to sandy soil (Table S1) which have high pH values (7.1–8.7).

About 134,473 ha of land in the Eastern Nile Delta was used to produce vegetables in 2017, this equivalent to 17.7% of all the absolute area used for vegetable production in Egypt. Tomato accounted for a further one-fifth (20.9%) of the total area. Potato is the second most cultivated vegetable, accounting for 14.6 of the vegetable area in the Eastern Nile Delta. Eggplant is produced in 9% of the vegetable area in the Eastern Nile Delta followed closely (8.3%) by pepper. The vegetables grown under high tunnels in the Eastern Nile Delta were dependent on areas planted in Dakahlia (32,534 tunnels), where cucumber is the main vegetable followed by pepper (EMARS, 2017). There are three growing seasons in this area: winter (from November to May), summer (from April/May to October), and Nili (from July/August to October).

The Damietta branch of the Nile River is the main source of freshwater for the irrigation of the most agricultural soil in the study area. More than 65% of the studied area depends on low-efficiency surface irrigation systems, which cause high water losses (Table S1).

### Sampling and analytical processing

Sixty sites were selected for different upper horizon (0–30 cm) of soil samples located in a rural area not close to possible industrial sites of pollution during October and November 2020. Soil samples were taken from the major vegetable production fields throughout the Eastern Nile Delta (Table S2 and Fig. 1). For each sampling site, five subsamples of soil consisting of one center and four corners were pooled and homogenized to form a composite sample that covered approximately 20 × 30 m^2^. The Global Positioning System (GPS) was used to determine the locations of samples on the spot (Fig. 1).

### Soil characterization

Soil samples were set in plastic bags and taken to the soil laboratory where they were air-dried for 2 weeks at room temperature (18–22 °C). Then the soil samples were sieved using a 2-mm aperture sieve and kept for more analyses. To estimate the particle-size distribution of investigated soil, the surface soil samples were conducted using the Pipette method according to Dewis and Freitas (1970). The pH was measured in 1:2.5 soil in deionized water at approximately 27 °C to solution ratio using Beckman glass electrode pH meter (Jackson, 1973). Electrical conductivity (EC) was detected in soil paste extract (Jackson, 1973), while the soil organic matter was measured by the titration method based on the oxidation of organic matter by K_2_Cr_2_O_7_ (Jackson, 1973). All selected soil samples were analyzed in triplicate, and the averages were calculated by three values.

### Digestion and estimation of trace metals

For trace metal determinations, the soil samples were oven-dried at 105 °C overnight, sieved mechanically using a 0.5-mm sieve, homogenized and then crushed to a 63-μm nylon mesh sieve according to the protocols described by Li et al. (2001). Subsequently, 1 g ± 0.01 of ground soil samples were weighed and digested in a Teflon bottles using nitric acid (HNO_3_) and perchloric acid (HClO_4_) mixture by the ratio of 4:1. Finally, the samples were heated at 40 °C for 1 h and augmented to 170 °C for 4 h till a clear solution was observed (Spalla et al., 2009). After filtration with Whatman No.1 filter paper, the acidic solution is diluted with deionized water to a total volume of 50 ml. The total heavy metal concentrations (mg kg^−1^ soil) were estimated using Thermo Scientific TM ICAPTM 7000 Plus Series ICP-OES (Ammann, 2007). Ultimately, to confirm the correctness of estimation, a certified reference material (CRM 1570) was occupied according to the National Institute of Standards and Technology Standard. Triplicate analyzed samples were laid out to keep the quality of the dealings, and the obtained results illustrated that all the estimated trace metals were analyzed with a 98.2% recovery level. The limits of quantification (LOQ) and detection (LOD) of the nine trace metals in the soil samples studied were 0.0033 and 0.001 mg kg^−1^ for soil, respectively.

### Assessment of soil pollution

The pollution indices of nine trace metals in the vegetable field soil from the Eastern Nile Delta were assessed by enrichment factor, geo-accumulation index, and contamination factor (Hakanson, 1980; Tomlinson et al., 1980).

### Enrichment factor (EF)

The enrichment factor (EF) of trace metals is based on the measured metal standardization against a reference metal. The reference metal should be of natural origin in the study area, and Fe was utilized as the normalization metal (Monged et al., 2020). The EF was calculated using Fe as the reference, according to the following equation:

1 $$\mathrm{EF}=\frac{\left(^{{C}_{n}}\big/_{{C}_{\mathrm{Fe}}}\right)\mathrm{ sample}}{\left(^{{C}_{n}}\big/_{{C}_{\mathrm{Fe}}}\right))\mathrm{ background}}$$

where (*C*_*n*_/*C*_Fe_) sample is the ratio of the concentration of each trace metal to the iron concentration in the soil sample, and (*C*_*n*_/*C*_Fe_) background is the ratio between the background value of trace metal and the background value of iron.

The soil pollution, based on the EF values was categorized into six groups, are listed in Table S3.

### Geo-accumulation index (Igeo)

The geo-accumulation index (Igeo) was used to estimate the metal load enrichment in the soil above the background level. It was calculated using the following equation that was offered by Muller (1969):

2 $$I\mathrm{geo}=\mathrm{Log}\left[\frac{{C}_{n}}{1.5\times {B}_{n}}\right]$$

where *C*_*n*_ was the trace metal *n* concentration in soil, and *B*_*n*_ is the background value of trace metal *n*. The background value is based on the world average value in shale (mg/kg) for the several metals under study (Cd = 0.3, Co = 19, Cr = 90, Cu = 45, Fe = 47,200, Mn = 850, Ni = 68, Pb = 20, and Zn = 95 mg kg^−1^) according to Turekian and Wedepohl (1961). The constant 1.5 was applied to balance the probable natural differences in the values of background that may be attributed to various lithologic effects (Stoffers et al., 1986).

Muller (1969) classifies the *I*geo value into seven categories (Table S3).

### Contamination factor (CF)

The contamination factor (CF) was computed using the following equation (Hakanson, 1980):

3 $$\mathrm{CF}=\frac{{C}_{n}}{{B}_{n}}$$

where *C*_*n*_ is the trace metal concentration in the soil and *B*_*n*_ is the value of the metal background. Four categories that are shown in Table S3 were proposed by Hakanson (1980) to classify the level of value of CF.

### Health risk assessment

In the present study, risk assessment parameters and methods reported by the United States Environmental Protection Agency (USEPA) were used to assess the non-carcinogenic and carcinogenic health risks of trace metals in soils. These risks were calculated through ingestion (*ing*), dermal (*derm*), and inhalation (*inh*) routes for adults and children.

The average daily intakes (ADIs) for each metal were estimated using the following equations (USEPA, 2002 and 2011):

4 $$\mathrm{ADI\ }ing=\frac{{C}_{s}\times {R}_{ing}\times \mathrm{EF}\times \mathrm{ED}}{\mathrm{BW}\times \mathrm{AT}}$$

5 $$\mathrm{ADI\ }derm=\frac{{C}_{s}\times \mathrm{SA}\times \mathrm{AF}\times \mathrm{ABS}\times \mathrm{EF}\times \mathrm{ED}}{\mathrm{BW}\times \mathrm{AT}}$$

6 $$\mathrm{ADI\ }inh=\frac{{C}_{s}\times {R}_{inh}\times \mathrm{EF}\times \mathrm{ED}}{\mathrm{PEF}\times \mathrm{BW}\times \mathrm{AT}}$$

where *C*_*s*_ is the trace metal concentration in the soil (mg kg^−1^). The other exposure parameters and their symbols, values, and units are presented in Table S4 in supplementary material (USEPA, 2002).

The non-carcinogenic risks were evaluated using hazard quotient (HQ) and hazard index (HI). The HQ value of each toxic metal for different exposure pathways is defined as Eq. (7) (Li et al., 2014; Rostami et al., 2019):

7 $$\mathrm{HQ}=\frac{\mathrm{ADI}}{RfD}$$

where *RfD* (mg k^−1^ day^−1^) is the reference dose of a trace metal that is listed in Table S5 in the supplementary material.

The HI is the summation of multiple exposure pathways of HQ for each trace metal:

8 $$\mathrm{HI}=\sum \mathrm{HQ}$$

If the obtained HQ or HI values are less than 1, there is no non-carcinogenic health risk. If these values exceed 1, there may be significant risk to human health.

The carcinogenic risks (CR) of trace metals in soil were computed by Eq. (9):

9 $$\mathrm{CR}=\sum \mathrm{ADI}\times \mathrm{SF}$$

where SF is the cancer slope factor of the trace metals. The values of SF are assumed in Table S5.

When CR is below 1 × 10^−6^, it is considered that there is no significant effect on the human body. CR between 1 × 10^−6^ and 1 × 10^−4^ is an acceptable range, and CR surpassing 1 × 10^−4^ is considered to be a large potential carcinogenic risk.

### Statistical analysis

Descriptive statistics related to the trace metal concentrations and soil properties were examined to explore the data. The data was checked for the frequency distribution by the Kolmogorov–Smirnov method. Pearson correlation analysis was performed to define the relationship between the soil variables. The cluster analysis was conducted to divide trace metals into different categories based on their sources in the soil of study.

Principal component analysis (PCA) was applied using factor extraction to identify the possible sources of nine trace metals in the study area. Components with eigenvalues higher than one unit after varimax rotation were taken (Borůvka et al., 2005; Hou et al., 2014). MSTAT-C, Microsoft Excel^®^ (2013) and Number Cruncher Statistical System (NCSS) statistical software were used for the data analyses. Maps were prepared with ArcGIS (version 10).

## Results and discussion

### The concentration of trace metals in vegetable field soils

The descriptive statistics of the content of trace metals in the vegetable field soil from the Eastern Nile Delta are given in Table 1. The descriptive statistics results show that the mean concentrations of Cd, Co, Cr, Cu, Fe, Mn, Ni, Pb, and Zn were 2.36, 16.12, 151.56, 9.85, 20,972.74, 479.63, 27.25, 28.31, and 100.01 mg kg^−1^, with a median concentration of 2.78, 19.98, 178.90, 10.48, 25,634.40, 576.30, 29.68, 31.30, and 116.91 mg kg^−1^, respectively. The SD values were high because the concentrations of trace metal showed quite high heterogeneous distribution in the studied area.

**Table 1:** Descriptive statistics of trace metal concentrations (mg kg^−1^) and soil properties (clay %, organic matter % (OM), pH, and electrical conductivity (EC) dS m^−1^) in the soils of the study area

Variables	Minimum	Q1	Median	Mean ± S.D	Q3	Maximum	CV %	Skewness	Kurtosis	K-S	MAC	Background values
Cd	0.17	2.06	2.78	2.36 ± 1	3.02	4.86	47.26	− 0.53	− 0.19	0.19	1–5	0.3
Co	1.65	4.32	19.98	16.12 ± 9	22.55	28.43	55.00	− 0.66	− 1.24	0.24	20–50	19
Cr	37.27	58.19	178.90	151.56 ± 70.16	205.24	255.05	46.29	− 0.60	− 1.22	0.21	50–200	90
Cu	2.09	5.86	10.48	9.85 ± 5	13.33	18.04	45.95	− 0.08	− 1.01	0.11	60–150	45
Fe	2162.20	6201.24	25,634.40	20,973 ± 11,553	29,016.30	38,350.88	55.09	− 0.70	− 1.13	0 .24	-	47,200
Mn	68.28	135.24	576.30	479.63 ± 267	677.72	935.98	55.67	− 0.53	− 1.23	0 .19	-	850
Ni	12.18	18.74	29.68	27.25 ± 8	33.06	40.04	29.02	− 0.33	− 1.23	0.15	20–60	68
Pb	0.00	17.11	31.30	28.31 ± 16	40.13	60.90	56.28	− 0.06	0.84	0.10	20–300	20
Zn	0.00	51.18	116.91	100.01 ± 41	126.76	170.30	41.15	− 0.62	− 0.50	0.18	100–300	95
Clay	0.70	8.15	38.40	31.17 ± 19	44.81	61.74	60.52	− 0.57	− 1.12	0.18	-	-
OM	0.42	0.83	1.14	1.21 ± 0.5	1.65	2.21	40.99	0.23	− 1.04	0.10	-	-
pH	7.10	7.80	8.10	8.02 ± 0.4	8.30	8.70	4.69	− 0.70	− 0.07	0.15	-	-
EC	0.98	1.42	1.85	2.07 ± 0.9	2.42	5.02	42.94	1.49	2.22	0.13	-	-

*K–S* Kolmogorov–Smirnov test, *CV* coefficient of variation, *BG* background as reported by Turekian and Wedepohl (1961), *Avg* average, *sd* standard deviation, *cv* coefficient of variation, *max* maximum value, *min* minimum value, *skew* skewness, *Q1* lower quartile, *Q3* upper quartile, *MAC* ranges of maximum allowable concentrations for trace metals in agricultural soils (mg kg^−1^) (Kabata-Pendias, 2010)

Omran (2016) found that the ranges of Co (70.5–113.8), Cu (7.7–280.3), Ni (61.3–88.7), and Zn (39.7–215.6) concentration (mg kg^−1^ soil) in the soil of Bahr El Baqar in the Eastern Nile Delta tended to be higher than the ranges found in this study, while the ranges of Cr (84.9–134.1) and Pb (16.4–52.4) tended to be lower. The mean concentrations of Co, Cr were higher than those from the Southwestern Nile Delta (Khalifa & Gad, 2018).

The slight difference between the lower quartile and the upper quartile points to the distribution and uniform source of trace metal in soil. Among the nine trace metals, Cd had a relatively slight difference between these quartiles. This may be due to adding more P-fertilizers that contain a significant amount of Cd, about 12.45 mg kg^−1^ (Salman et al., 2017). Moreover, cadmium may also be added to soils adjacent to roads (Li et al., 2018).

Among all trace metals, Fe has the largest mean value (20,973 mg kg^−1^), while Cd has the lowest mean value of 2.36 mg kg^−1^. These results were consistent with the mean values of Fe and Cd found in agricultural soil in Egypt (EL-Bady & Metwally, 2019). The mean concentrations of Cd, Co, Cr, Cu, Ni, Pb, and Zn were below the maximum value of the range of maximum allowable concentrations (MAC) mentioned by Kabata-Pendias (2010). The mean concentrations of Co, Cu, Fe, Mn, and Ni were lower than their corresponding background values. However, the mean concentrations of Cd, Cr, Pb, and Zn exceeded their background values, which can demonstrate that the soil of the studied area is polluted with these metals by anthropogenic activities (Khalifa & Gad, 2018).

In the same line, higher contents of Cd, Pb, Cu, and Zn were observed in vegetable fields in China (Kuerban et al., 2020). The main contribution of trace metals to agricultural soil is the use of chemical fertilizers, pesticides, and organic fertilizers (Kuerban et al., 2020; Mo et al., 2020; Omran, 2016). Thus, Cd, Co, Pb, and Zn will require more attention in the future, and continuous monitoring of the application of agrochemicals in the production of vegetables is recommended to decrease the probable ecological risks caused by these trace metals in the soil of the Eastern Nile Delta.

The coefficient of variation (CV) measures the variability degree of the trace metal concentrations in soil. A high value of CV indicates that the contamination of soil by trace metal is produced mainly from human activities. A low CV value indicates that trace metal pollution is due to natural sources (Cai et al., 2015; Keshavarzi & Kumar, 2020; Orellana et al., 2020). The high concentrations of metals, coupled with high variation, suggest that anthropogenic inputs may be their primary source in the study area. The highest CV values were recorded for Cd (47.26), Co (55.00), Cr (46.29), Cu (45.95), Fe (55.09), Mn (55.53), Pb (56.28), and Zn (41.15). The high CV value confirms that these metals have a wide concentration range and is an indication of the potential impact of human activities on them. Lower values of CV for Ni (29.02) confirm that human activities are less effective on it (Cai et al., 2015).

The skewness values of Cu and Pb were near zero and came near a normal distribution, while the other metals had slightly negative skewness, indicating that the center of distribution is shifted to the right. The kurtosis of Pb was greater than zero, indicating that the distribution has a towering shape. The kurtosis values of other elements were negative, indicating that the distribution has a flat shape. The results of the Kolmogorov–Smirnov test (*p* < 0.05) confirm that the concentrations of Cu, Ni, and Pb have a nearly normal distribution, while the concentrations of other metals did not follow a normal distribution.

### Soil physicochemical parameters

The descriptive statistical analyses of the physicochemical parameters of soil, i.e., clay, OM, pH, and EC are given in Table 1 and show that the study area had a wide range of contents of clay (0.70–61.74; median: 38.40) and EC (0.98–5.02; median: 1.85 dS m^−1^). The soil was moderately alkaline with mean pH of 8.02, with low OM (mean 1.21), as typically found in Mediterranean environments. The CV values were highest for clay (60.52%), OM (40.99%), and EC (42.94%), indicating a non-homogenous distribution. The soil pH had a homogeneous distribution of variables, as indicated by the low value of CV of 4.69%. These results are in line with those found by Itta et al. (2019).

The soil clay and pH observations skewed to the right, while the OM and EC observations skewed to the left. The kurtosis results showed that clay and OM parameters revealed a flat shape, while the distribution of EC values exhibited a towering shape. The pH observations were subject to normal distributions. The K-S test suggested that clay values were not normally distributed, while OM, pH, and EC values approached a normal distribution.

### Multivariate statistical analysis

#### Correlation analysis

The correlation between different variables is presented in Table 2. The relationship that occurs between trace metals in the soil is usually due to parent material, the influence of pedogenic process, and the effect of human activities (Cheng et al., 2015). Very significant positive correlations were found between all trace metals except Cu, which indicated that Cd, Co, Cr, Fe, Mn, Ni, Pb, and Zn could be largely derived from the same sources and spreading (Cheng et al., 2015). The Cu concentration was positively and significantly correlated with the concentration of Fe, Mn, and Ni. Non-significant correlations of Cu with Cd, Co, Pb, and Zn may be due to the various processes like external inputs and biological effects. A similar trend was reported in the Southwestern Nile Delta, where the associations between Cr, Cu, Ni, Pb, and Zn and scavenger metals (Fe and Mn) had significant high associations (Khalifa & Gad, 2018).

**Table 2:** The Pearson correlation coefficients between trace metals and soil properties

Variables	Cd	Co	Cr	Cu	Fe	Mn	Ni	Pb	Zn	Clay	OM	pH
Cd	1											
Co	0.780**	1										
Cr	0.763**	0.957**	1									
Cu	0.097	0.178	0.202*	1								
Fe	0.804**	0.986**	0.951**	0.216*	1							
Mn	0.735**	0.979**	0.953**	0.201*	0.982**	1						
Ni	0.665**	0.942**	0.905**	0.202*	0.948**	0.952**	1					
Pb	0.576**	0.664**	0.668**	− 0.017	0.579**	0.601**	0.496**	1				
Zn	0.589**	0.676**	0.665**	0.161	0.651**	0.614**	0.583**	0.655**	1			
Clay	0.732**	0.895**	0.880**	0.212*	0.905**	0.895**	0.816**	0.634**	0.709**	1		
OM	0.556**	0.774**	0.763**	0.321**	0.777**	0.775**	0.796**	0.504**	0.549**	0.688**	1	
pH	0.288**	0.410**	0.483**	− 0.059	0.396**	0.402**	0.356**	0.497**	0.510**	0.531**	0.345**	1
EC	0.371**	0.400**	0.455**	0.017	0.387**	0.416**	0.359**	0.456**	0.240*	0.320**	0.251*	0.365**

^*^p < 0.05, **p < 0.01

The correlation between trace metal concentrations and soil properties is presented in Table 2. Clay % had very significant positive correlations with Cd (0.732), Co (0.895), Cr (0.880), Fe (0.905), Mn (0.895), Ni (0.816), Pb (0.634), and Zn (0.709) and had significant positive correlations with Cu (0.212). The high degree of correlation endorses that the clay is acting as a metal carrier and plays a vital role in the distribution pattern of trace metals (Khalifa & Gad, 2018). The positive relationships between all studied trace metals and organic matter percentages were very highly significant. The soil properties, especially soil organic matter and clay particle content, effectively absorb trace metals (Khalifa & Gad, 2018). Soil organic matter is one of the main soil properties with dual effects on trace metals mobility (Mo et al., 2020). This may be because organic matter decomposition produces organic chemicals into soil solution that might act as chelates and raise the bioavailability of trace metals. However, the organic matter of the clay fraction might also decrease the trace metal bioavailability by forming stable complexes with humic substances or through adsorption (Dabkowska-Naskret, 2003). Both pH and EC correlated positively with Cd, Co, Fe, Mn, Ni, Pb, and Zn. Significant correlations were not found between Cu and soil pH and EC. A similar finding has been found by Itta et al. (2019).

#### Factor analysis

Factor analysis was carried out to identify the sources of pollution. The results of factor analysis using the varimax rotation method are presented in Table 3. Four factors with eigenvalues > 1.0 were selected for the retention data of trace metals in the studied area which contributed to approximately 87.41% of the total variance. The first factor explains 50.43% of the total variance with negative loadings on all elements. The first factor was characterized by high loadings (≥ 0.72) of Co, Cr, Fe, Mn, and Ni and moderate loading (0.520) of Cd. Several studies found that most of these metals originated from parent material in agricultural soil (Cheng et al., 2015). Thus, this factor may represent a natural source. Factor 2 accounts only for about 11.50% of the total variability. It was heavily loaded (≥ 0.99) with Cu with positive loading. The Cu level in the soil is most probably due to agricultural activities such as fertilizer, fungicide application, and irrigation with wastewater (Zhang et al., 2018; Zhao et al., 2019). Factor 3 explained 13.28% of the total variance. It mainly condensed the information of Pb with negative loadings (− 0.867). The higher content of Pb might have come from anthropogenic activities (Orellana et al., 2020). The fertilizers and traffic emissions can be the major source of the amount and distribution of Pb in agricultural soils (Cai et al., 2015). Factor 4 exhibits 12.20% of the total variance with positive loading (0.852) on Zn. Organic manure and phosphate fertilizers cause a significant expansion in the levels of Zn (Marrugo-Negrete et al., 2017).

**Table 3:** Factor loadings rotated matrix for trace metals

Variables	Factor 1	Factor 2	Factor 3	Factor 4
Cd	− 0.520	0.017	− 0.227	0.217
Co	− 0.876	0.065	− 0.287	0.250
Cr	− 0.847	0.093	− 0.305	0.232
Cu	− 0.120	0.991	0.028	0.051
Fe	− 0.885	0.130	− 0.186	0.233
Mn	− 0.915	0.087	− 0.241	0.186
Ni	− 0.942	0.081	− 0.128	0.200
Pb	− 0.346	− 0.051	− 0.867	0.278
Zn	− 0.377	0.078	− 0.302	0.852
Eighen value	4.54	1.035	1.20	1.10
Variance %	50.43	11.50	13.28	12.20
Cumulative (%)	50.43	61.93	75.21	87.41

The spatial and vertical distribution of trace metal levels is influenced by soil properties, for example, the content of clay and OM. Industrial activity, agricultural practices, and intensive urbanization are the primary anthropogenic sources of trace metal pollution. Cr, Cu, Ni, Pb, and Zn are derived from uncontrolled utilization of phosphate fertilizers and by atmospheric deposition from industrial activity and urban areas (Chen et al., 2018; Orellana et al., 2020). Co is enhanced by the organic manure application to the agricultural soil (Omran, 2016). Cu and Cr are produced from reactions involving Cu SO_4_, which mainly originated from disease prevention (Zhang et al., 2018; Zhao et al., 2019). The results of factor analysis also indicate that Fe and Mn came from different potential sources. Agricultural soil can be polluted with Co, Cr, Ni, and Zn that might have originated mainly from the application of fertilizers and organic manure (Kabata-Pendias, 2010; Omran, 2016). Moreover, it can be polluted with Cr, Cu, Ni, Pb, and Zn that result from industrial activity and atmospheric deposition (Darwish & Pöllmann, 2015). Atmospheric deposition from industrial and urban areas represented 80.36% of Zn, 76.55% of Pb, 67.48% of Cu, 62.23% of Cr, and 37.79% of Ni entering agrarian soil from anthropogenic activities (Chen et al., 2018). It appears that anthropogenic and lithologic practices are the chief sources of Cd accumulation in soil. Cadmium is a metal marker of agricultural production activity, and it can be coming from atmospheric deposition (Kabata-Pendias, 2010; Zhang et al., 2018).

#### Hierarchical cluster analysis

**Fig. 2:**
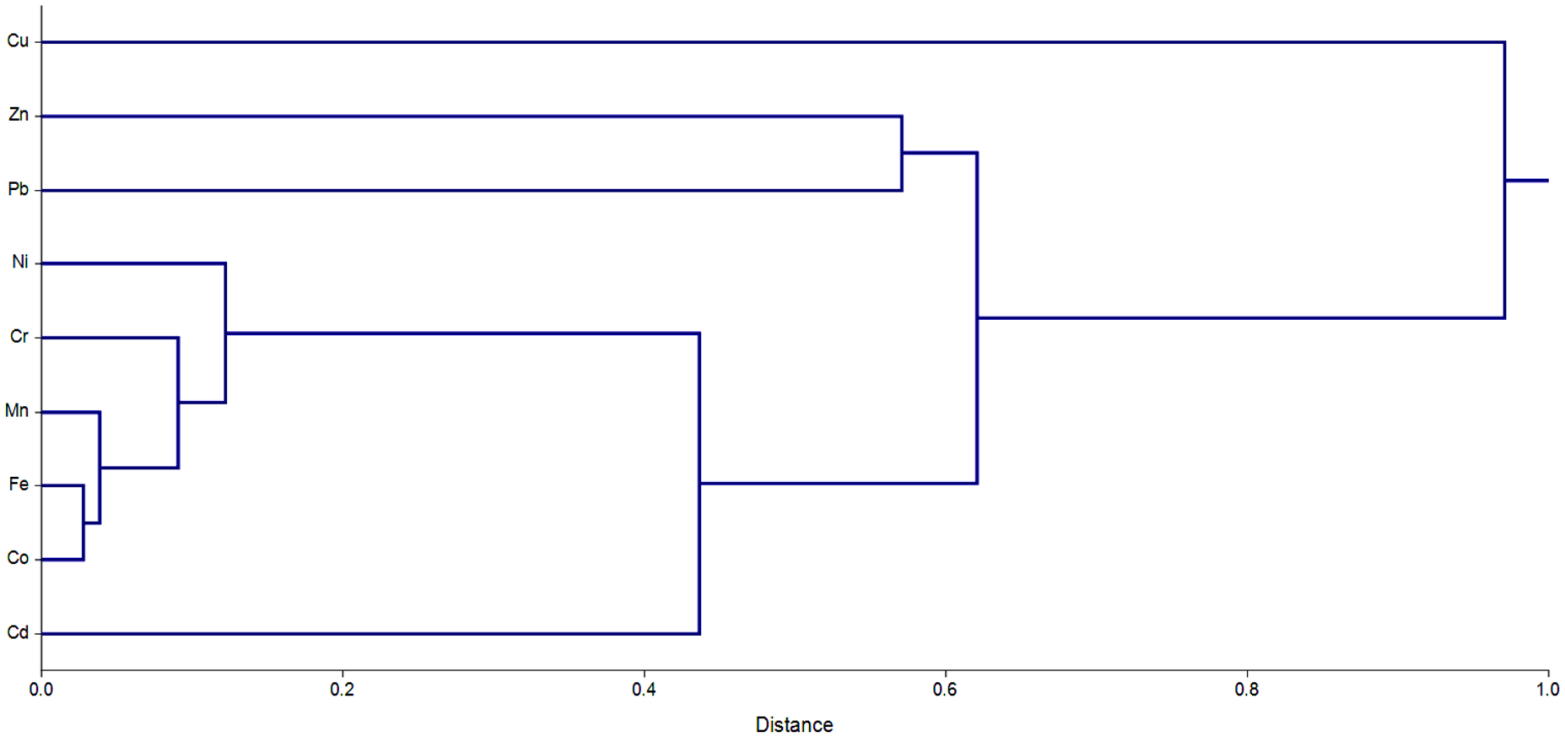
Hierarchical clustering of trace metals in vegetable soil in the Eastern Nile Delta

The results of the hierarchical cluster analysis show that nine trace metals in the soil of the Eastern Nile Delta were divided into five categories (Fig. 2). The first cluster contained Cu. The second cluster contained Zn. Cluster three contained Pb. The fourth cluster contained Cd. The fifth cluster contained Mn, Fe, Co, and B. The clustering results agreed with the factor analysis results.

### Spatial distribution of trace metals

**Fig. 3:**
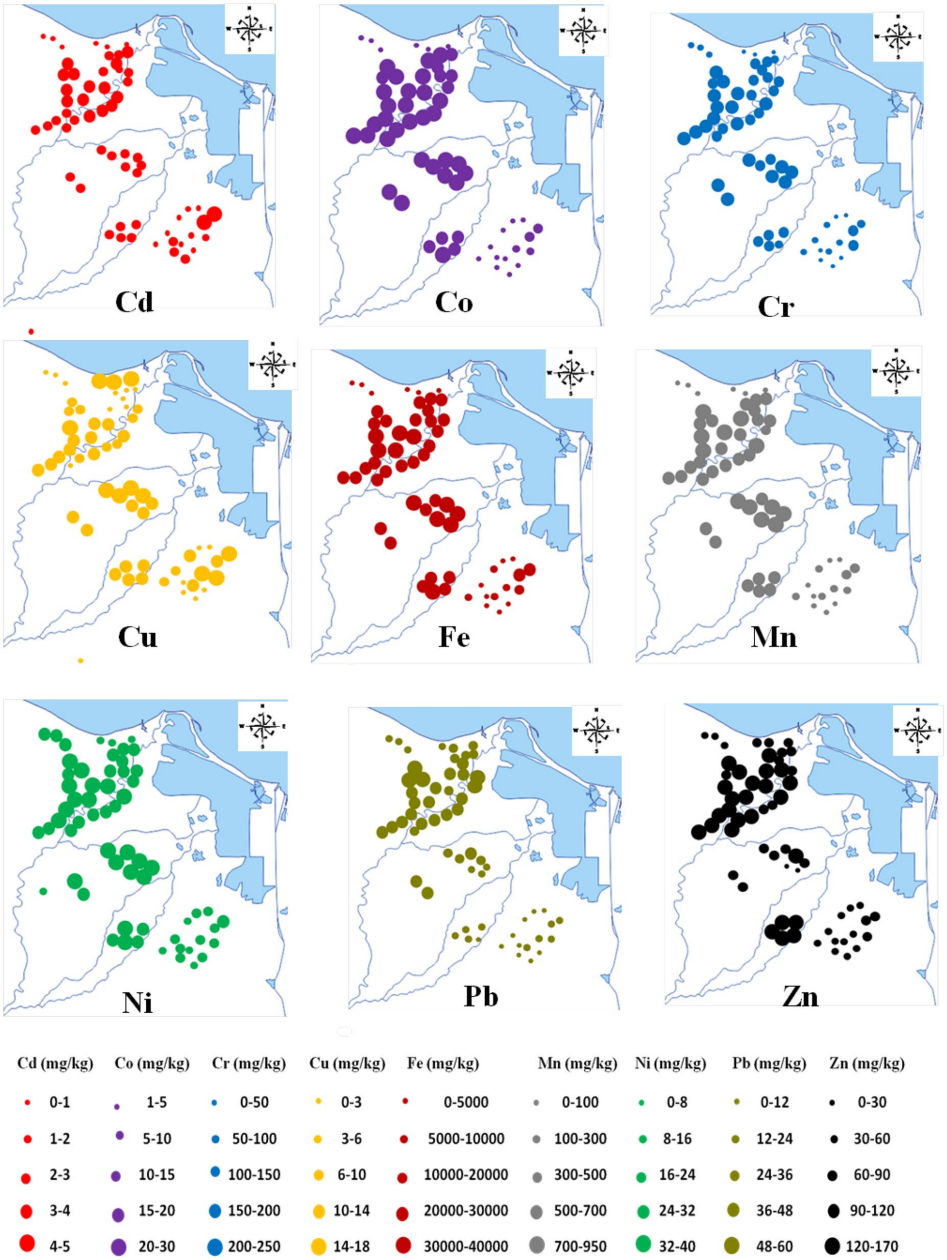
Spatial distribution maps of trace metals in the samples of vegetable field soil from the Eastern Nile Delta

The spatial distribution maps of trace metal contents in the vegetable field soil of the Eastern Nile Delta are presented in Fig. 3. Generally, the concentrations of all studied trace metals were the highest in the northwest of the region (i.e., Kafr Saad, Sherbin, Belkas, Talkha, Fariskur, and Elzarka). The possible reason for this is that some vegetable fields are close to industrial regions and intensive traffic activity and irrigated with wastewater which leads to higher soil trace metal contents. Additionally, very high values of Co, Cr, Cu, Fe, Mn, and Ni were also recorded in the central part (i.e., Bani Ebeid, Awlad Saqr, Al-Sembelawaan, Abu Kabir, and Fakoos). The sites with the lowest concentration of most trace metals are located in the southeast of the region (i.e., New Salhia and Qasasin Alsharq) which suggests that vegetable soils in this part might not have been affected by industrial activities. The distribution patterns of the Cd and Cu are mainly similarly distributed over the study region. This suggests the primary role of agricultural activities, such as fertilizer and pesticide application in the vegetable fields, as the main pollutant sources.

### Pollution assessment of trace metals

#### Enrichment factor (EF)

**Fig. 4:**
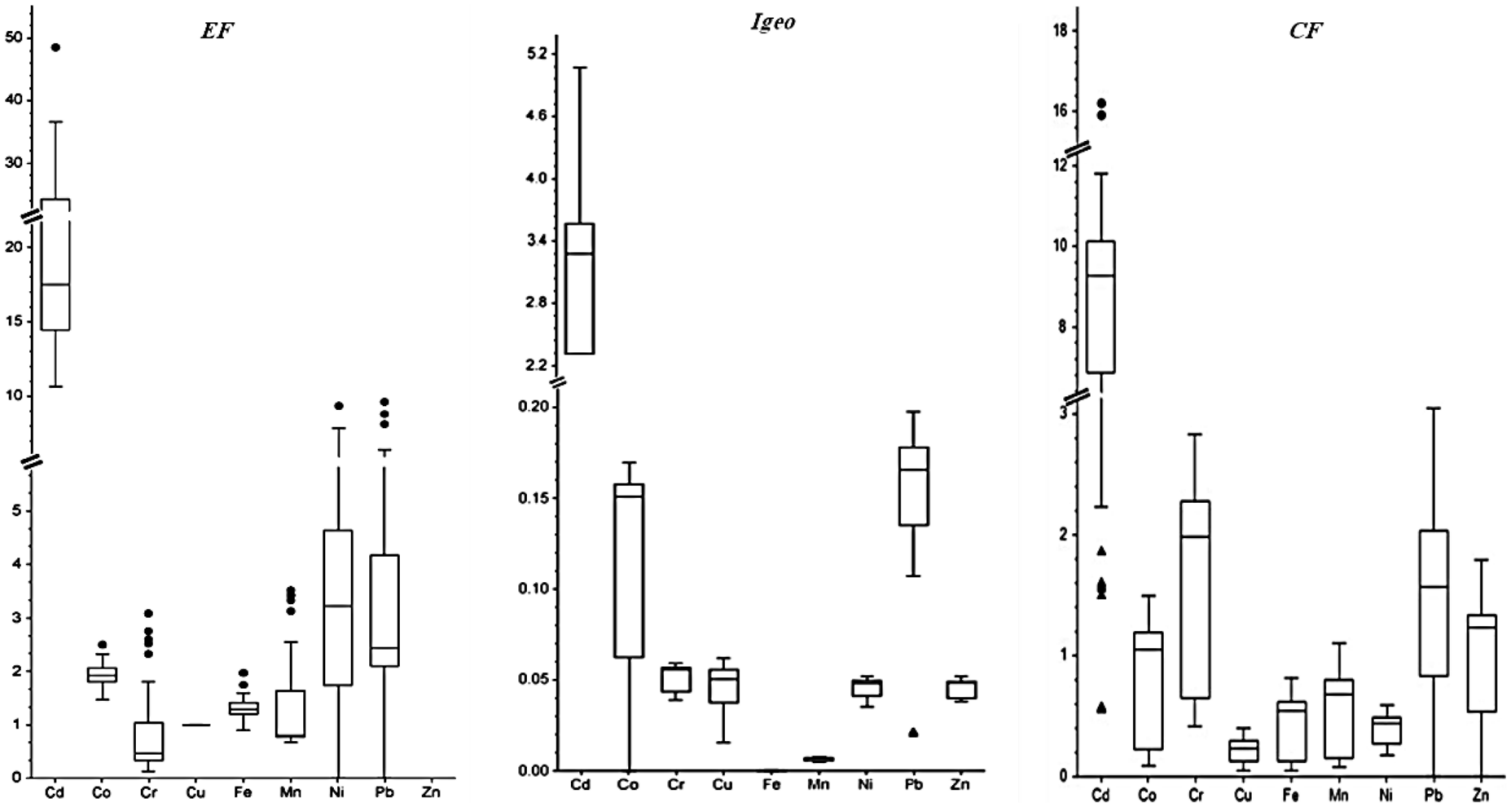
Box-whisker plots of the EF, *I*geo, and CF of trace metals in soils (the whisker shows the minimum and maximum values and the line of each plot is the median value)

The EF values for the studied metals are presented in Fig. 4. The EF values indicated that Co, Cu, Fe, Mn, and Ni < 2, show no enrichment. The metals of Cr, Pb, and Zn indicated moderate enrichment with EF mean of 4.856, 4.773, and 3.874 respectively. Khalifa and Gad (2018) and Abou El-Anwar (2019) found similar results. The maximum EF value was 22.687 for Cd and consequently signifies very high enrichment. The EF values < 2 point to the trace metal completely coming from a geological origin, but the EF values > 2 indicate that the trace metal possibly derives from anthropogenic activities (Saha et al., 2017).

#### Geo-accumulation index (Igeo)

The values of the geo-accumulation index (*I*geo) for the studied metals are illustrated in Fig. 4. Generally, the positive *I*geo values indicate that the metal contamination is related to anthropogenic activities (Rajmohan et al., 2014). The mean values of *I*geo increased in the order of Fe (0.0002) < Mn (0.007) < Zn (0.0450) < Cu and Ni (0.046) < Co (0.123) < Pb (0.145) < Cd (2.047) < Cr (3.114). The range of *I*geo values for individual metal is as follows: Cr (0.039–0.059), Cd (5.705–5.068), Co (0–0.169), Cu (0.016–0.062), Fe (0.00016–0.00022), Mn (0.005–0.008), Ni (0.035–0.052), Pb (− 0.012–0.198) and Zn (0–0.052). Based on the mean values of *I*geo, Cr had trace contamination, and Cd had moderately to heavily contaminated. However, the other metals showed uncontaminated to moderately pollution load. This result is consistent with one previous review on agricultural soil which shows that the *I*geo values of Cr and Cd were above 1 (Castro-González et al., 2017; Khalifa & Gad, 2018; Zhang et al., 2018).

#### Contamination factor (CF)

The computed results of the contamination factor (CF) for studied trace metals are presented in Fig. 4. The mean CF values of trace metals decreased in the following order: Cd (7.851) > Cr (1.684) > Pb (1.416) > Zn (1.053) > Co (0.848) > Mn (0.564) > Fe (0.444) > Ni (0.401) > Cu (0.219). The mean CF value for Cd indicated a very high contamination level (CF > 6), while the mean CF values for Cr, Pb, and Zn showed a moderate contamination level (1 < CF < 3); and the mean CF values for Co, Mn, Fe, Ni, and Cu pointed to a low contamination level (CF < 1). CF values show minor similarity with Omran’s (Omran, 2016) study on the soil of Bahr El Baqar in the Eastern Nile Delta, Shokr et al. (2016) in the soil of the middle Nile Delta and Abou El-Anwar (2019) in the soil of the Upper of Egypt.

#### Health risk assessment

The values of *HQ*, *HI*, and *CR* related to nine metals for both adults and children are summarized in Table 4. The *HQ* values of different metals through three pathways were reduced in the order of ingestion > dermal absorption > inhalation. This result implies that ingestion of soil particles is the major route for trace metals that were adverse to human health. Previous studies have obtained similar results (Yang et al., 2018; Zhao et al., 2019). The HI values of soil trace metals for both adults and children were far lower than the safe level (HI ≤ 1) and reduced in the following order: Zn > Cr > Mn > Fe > Cd > Pb > Ni > Co > Cu. These results indicated that the non-carcinogenic threat for children and adults is relatively light across the vegetable field soil of the study area.

**Table 4:** Health risks of trace metals in vegetable soils from the Eastern Nile Delta

	Hazard quotient (ingestion)					Hazard quotient (dermal)			Hazard quotient (inhalation)			Hazard index			Carcinogenic risks	
	Adults			Children		Adults	Children		Adults	Children		Adults	Children		Adults	Children
Cd			1.61E-03	3.61E-03		1.29E-03	8.43E-03		3.44E-05	8.30E-05		2.93E-03	1.21E-02		2.17E-09	5.23E-09
Co			5.52E-04	1.24E-03		5.51E-06	3.61E-05		4.12E-04	9.95E-04		9.70E-04	2.27E-03		2.31E-08	5.57E-08
Cr			3.46E-02	7.75E-02		1.38E-02	9.04E-02		7.74E-04	1.87E-03		4.92E-02	1.70E-01		9.30E-07	2.24E-06
Cu			1.69E-04	3.78E-04		4.49E-06	2.94E-05		3.58E-08	8.64E-08		1.73E-04	4.07E-04		-	-
Fe			2.05E-02	4.60E-02		1.64E-04	1.07E-03		3.83E-06	9.24E-06		2.07E-02	4.70E-02		-	-
Mn			7.14E-03	1.60E-02		1.42E-03	9.28E-03		4.90E-03	1.18E-02		1.35E-02	3.71E-02		-	-
Ni			9.33E-04	2.09E-03		2.96E-05	1.94E-04		1.93E-07	4.66E-07		9.63E-04	2.28E-03		3.34E-09	8.07E-09
Pb			5.54E-03	1.24E-02		2.95E-04	1.93E-03		1.17E-06	2.84E-06		5.84E-03	1.43E-02		1.65E-07	3.70E-07
Zn			2.28E-04	5.11E-04		9.11E-06	5.97E-05		4.87E-08	1.18E-07		9.42E-02	2.85E-01		-	-

The HI and CR values for adults were lower than the values for children. Previous studies reported that children experienced higher hazards by trace metal contamination than adults (Zhao et al., 2019). The CR values of Cd, Cr, Ni, and Pb were lower than the safe value (1 × 10^−6^) and had no risk (El-Alfy et al., 2017). The CR values for Cr were between 1 × 10^−6^ and 1 × 10^−4^ for children, which indicate a lower but elevated carcinogenic risk. Therefore, children have much more chances of carcinogenic risk from Cr exposure in the study area than adults. Similar results were found by Song et al. (2018), Mo et al. (2020), and Liu et al. (2021) who reported that Cr posed a significant carcinogenic risk. Moreover, previous studies have revealed that human exposure to low Cr concentrations for the long term can cause poisonous and cancer-causing impacts in people (Khan et al., 2015). Also, Zhao et al. (2019) and Liu et al. (2021) reported the Cr in soil caused a significant carcinogenic risk to adults and children. Thus, particular attention should be paid to Cr pollution. Risks associated with non-carcinogenic and cancer results from the investigation region were generally lower than those found in the rural areas irrigated with wastewater in the Nile Delta (El-Alfy et al., 2017).

## Conclusions

This is the first study of risk assessment of the pollution of vegetable field soil by trace metals in Egypt. The results showed that the overall quality of vegetable fields in the Eastern Nile Delta is relatively safe although some samples reveal serious pollution problems by Cd and Cr. The mean contents of Cu, Mn, and Ni in soil samples were lower than their corresponding background concentrations, while the mean values of Cd, Co, Pb, and Zn exceeded their background values. The high concentrations of metals, coupled with high variation, can demonstrate that anthropogenic activities may be their primary source in the study region. The determined enrichment factor, geo-accumulation index, and contamination factor revealed the study soil experienced low to moderate pollution and the Cd and Cr pollution was very serious. The hazard index values from nine trace metals through the three exposure pathways for both adults and children were in an acceptable range and far lower than the safe level demonstrating that there was not a non-carcinogenic hazard for these age groups. The carcinogenic risk of Cd, Co, Ni, and Pb metals was within the acceptable range for both adults and children, while Cr had a significant carcinogenic health risk to children in the study area. Further studies are required to assess the health risk of trace metal intake from vegetables. The study highlights the importance of monitoring trace metals in vegetable field soil, enhancing soil management, decreasing the application of chemical fertilizers and pesticides, and irrigating with fresh water as excellent solutions for the long term to reduce further soil pollution in the vegetable field soil of the Eastern Nile Delta region.

## Supplementary Information

Below is the link to the electronic supplementary material.

Supplementary file1 (DOCX 40 KB)

## Data Availability

The datasets used and analyzed during this article are available from the corresponding author on reasonable request.
