# Supplementary material for: Pollution and health risk assessment of trace metal in vegetable field soils in the Eastern Nile Delta, Egypt
Source: Environ Monit Assess. 2022 Jun 29;194(8):540. doi: 10.1007/s10661-022-10199-1 (PMC9242902; doi:10.1007/s10661-022-10199-1)
Supplement: Supplementary file 1 — Supplementary file1 (DOCX 40 KB) [file 10661_2022_10199_MOESM1_ESM.docx]

**Pollution and health risk assessment of trace metal in vegetable field soils in the Eastern Nile Delta, Egypt**

Ehab A. Ibrahim^a*^ . El-Metwally M. Selim^b^

^a^ *Cross Pollinated Vegetable Crops Research Department, Horticulture Research Institute, Agricultural Research Center, 9 Cairo University St., Orman, Giza, Egypt*

^b^ *Department of Soil Sciences, Faculty of Agriculture, Damietta University, Damietta 34517, Egypt*

^*^ Corresponding author.

*E-mail addresses:* e_ebraheem@yahoo.com (E. A. Ibrahim)

ORCID [0000-0003-1001-2673](javascript:popup_orcidDetail('https://orcid.org','0000-0003-1001-2673');)

**Table S1** Soil texture class and irrigation system in the studied sites

| Site | Soil texture | Irrigation system | Site | Soil texture | Irrigation system | Site | Soil texture | Irrigation system |
| --- | --- | --- | --- | --- | --- | --- | --- | --- |
| 1 | Sand | Furrow | 21 | Clay | Furrow | 41 | Clay | Furrow |
| 2 | Loamy sand | Drip | 22 | Clay | Furrow | 42 | Clay-loam | Furrow |
| 3 | Loamy sand | Furrow | 23 | Clay | Furrow | 43 | Clay-loam | Furrow |
| 4 | Sandy clay loam | Furrow | 24 | Clay | Furrow | 44 | Sandy clay loam | Furrow |
| 5 | Clay-loam | Furrow | 25 | Clay | Furrow | 45 | Clay | Drip |
| 6 | Sand | Drip | 26 | Clay | Furrow | 46 | Clay | Furrow |
| 7 | Sand | Drip | 27 | Clay | Furrow | 47 | Clay | Furrow |
| 8 | Sand | Drip | 28 | Clay | Furrow | 48 | Clay | Furrow |
| 9 | Sand | Drip | 29 | Clay | Furrow | 49 | Clay | Furrow |
| 10 | Sand | Drip | 30 | Clay | Furrow | 50 | Sand | Drip |
| 11 | Sand | Drip | 31 | Clay | Furrow | 51 | Sand | Furrow |
| 12 | Sand | Drip | 32 | Clay | Furrow | 52 | Sand | Drip |
| 13 | Sand | Drip | 33 | Clay | Furrow | 53 | Sand | Drip |
| 14 | Sand | Drip | 34 | Clay | Furrow | 54 | Sand | Drip |
| 15 | Clay | Furrow | 35 | Clay-loam | Furrow | 55 | Sand | Drip |
| 16 | Clay | Furrow | 36 | Clay-loam | Furrow | 56 | Clay-loam | Drip |
| 17 | Clay | Furrow | 37 | Clay-loam | Furrow | 57 | Clay-loam | Drip |
| 18 | Clay | Furrow | 38 | Clay-loam | Furrow | 58 | Clay-loam | Drip |
| 19 | Clay | Furrow | 39 | Clay-loam | Furrow | 59 | Clay-loam | Drip |
| 20 | Clay | Furrow | 40 | Clay-loam | Furrow | 60 | Clay-loam | Drip |

**Table S2** Sampling site, distric, type of grown vegetable crop and farming system

| Site | Distric | Crop | Farming system | Site | Distric | Crop | Farming system | Site | Distric | Crop | Farming system |
| --- | --- | --- | --- | --- | --- | --- | --- | --- | --- | --- | --- |
| 1 | Qasasin Alsharq | beans | Open-field | 21 | Awlad Saqr | potato | Open-field | 41 | Sherbin | peas | Open-field |
| 2 | Qasasin Alsharq | Strawberry | Open-field | 22 | Awlad Saqr | potato | Open-field | 42 | Sherbin | cabbage | Open-field |
| 3 | Qasasin Alsharq | cabbage | Open-field | 23 | Awlad Saqr | carrot | Open-field | 43 | Belkas | beans | Open-field |
| 4 | Fakoos | squash | Open-field | 24 | Bani Ebeid | cabbage | Open-field | 44 | Kafr El-Batteikh | eggplant | Open-field |
| 5 | Fakoos | tomato | Open-field | 25 | Bani Ebeid | beans | Open-field | 45 | Kafr Saad | potato | Open-field |
| 6 | New Salhia | Strawberry | Open-field | 26 | Bani Ebeid | peas | Open-field | 46 | Kafr Saad | potato | Open-field |
| 7 | New Salhia | squash | Open-field | 27 | Al-Sembelawaan | peas | Open-field | 47 | Kafr Saad | cabbage | Open-field |
| 8 | New Salhia | eggplant | Open-field | 28 | Al-Sembelawaan | potato | Open-field | 48 | Kafr Saad | pepper | Open-field |
| 9 | New Salhia | tomato | Open-field | 29 | Sherbin | potato | Open-field | 49 | Kafr Saad | pepper | Open-field |
| 10 | New Salhia | pepper | Open-field | 30 | Elzarka | carrot | Open-field | 50 | Kafr El-Batteikh | eggplant | Open-field |
| 11 | New Salhia | eggplant | Open-field | 31 | Elzarka | sweet potato | Open-field | 51 | Kafr El-Batteikh | squash | Open-field |
| 12 | New Salhia | pepper | Open-field | 32 | Elzarka | peas | Open-field | 52 | Kafr El-Batteikh | potato | Open-field |
| 13 | Qasasin Alsharq | squash | Open-field | 33 | Fariskur | potato | Open-field | 53 | Belkas | tomato | Open-field |
| 14 | Qasasin Alsharq | tomato | Open-field | 34 | Fariskur | squash | Open-field | 54 | Belkas | squash | Open-field |
| 15 | Fakoos | peas | Open-field | 35 | Talkha | potato | Open-field | 55 | Belkas | tomato | Open-field |
| 16 | Abu Kabir | peas | Open-field | 36 | Talkha | beans | Open-field | 56 | Belkas | cucumber | High tunnel |
| 17 | Abu Kabir | tomato | Open-field | 37 | Talkha | potato | Open-field | 57 | Belkas | cucumber | High tunnel |
| 18 | Abu Kabir | pepper | Open-field | 38 | Talkha | cabbage | Open-field | 58 | Belkas | pepper | High tunnel |
| 19 | Fakoos | cabbage | Open-field | 39 | Belkas | carrot | Open-field | 59 | Mansoura | cucumber | High tunnel |
| 20 | Awlad Saqr | carrot | Open-field | 40 | Sherbin | potato | Open-field | 60 | Mansoura | cucumber | High tunnel |

**Table S3** The criteria of pollution grade for *Igeo*, *EF* and *CF* of trace metals in the study area soils

| *Igeo* | Pollution level | *EF* | Pollution level | *CF* | Pollution level |
| --- | --- | --- | --- | --- | --- |
| *Igeo*≤0 | Uncontamination | *EF* < 1 | No enrichment | *CF*≤1 | Low contamination |
| 0<*Igeo*≤1 | Uncontamination to moderate contamination | (1–2) | Low degree | 1<*CF*≤3 | Moderate contamination |
| 1<*Igeo*≤2 | Moderate contamination | (2–5) | Moderate degree | 3<*CF*≤6 | Considerable contamination |
| 2<*Igeo*≤3 | Moderate to heavy contamination | (5–20) | Considerable degree | *CF*>6 | Very high contamination |
| 3<*Igeo*≤4 | Heavy contamination | (20–40) | Very high degree |  |  |
| 4<*Igeo*≤5 | Heavy to extreme contamination | *EF* > 40 | Exceedingly high degree |  |  |
| *Igeo*>5 | Extreme contamination |  |  |  |  |

**Table S4** Input parameters to describe the average daily intake of trace metals through different exposure pathways [38-40].

| Parameters | Symbols | Value | |  | Units |
| --- | --- | --- | --- | --- | --- |
|  |  | Adults | Children |  |  |
| Ingestion rate | *ADIing* | 100 | 200 |  | mg/day |
| Dermal absorption rate | *ADIderm* |  |  |  | mg/day |
| Inhalation rate | *ADIinh* | 14.5 | 7.5 |  | mg/day |
| Exposure frequency | *EF* | 350 | 350 |  | Days/year |
| Exposure duration | *ED* | 24 | 6 |  | Years |
| Exposed skin area | *SA* | 5075 | 2448 |  | cm^2^ |
| Skin adherence factor | *AF* | 0.07 | 0.2 |  | mg/m^2^·day |
| Dermal absorption factor | *ABS* | 0.001 | 0.001 |  | - |
| Particle emission factor | *PEF* | 1.36E+09 | 1.36E+09 |  | m^3^/kg |
| Average exposure time | *AT* | non-carcinogens = *ED*×365  carcinogens = 25550 | |  | days |
| Average bodyweight | *BW* | 70 | 15 |  | kg |

**Table S5** Reference dose (*Rfd*) and slope factor (*SF*) values used to evaluate the risk assessment of trace metals

| Metals | *Rfd* (mg kg^-1^ day^-1^) | | |  | *SF* **(**mg kg^-1^ day^-1^) | |
| --- | --- | --- | --- | --- | --- | --- |
|  | Ingestion | Dermal absorption | Inhalation |  | Ingestion | Inhalation |
| Cd | 1.00E-03 | 1.00E-03 | 1.00E-03 |  |  | 6.30E+00 |
| Co | 3.00E-03 | 3.00E-03 | 3.00E-03 |  |  |  |
| Cr | 3.00E-03 | 6.00E-05 | 2.86E-05 |  | 5.00E-01 | 4.20E+01 |
| Cu | 4.00E-02 | 4.00E-02 | 4.00E-02 |  |  |  |
| Fe | 7.00E-01 | 7.00E-01 | 7.00E-01 |  |  |  |
| Mn | 1.40E-02 | 1.40E-02 | 1.40E-02 |  |  |  |
| Ni | 2.00E-02 | 2.00E-02 | 2.00E-02 |  |  | 8.40E-01 |
| Pb | 3.50E-03 | 3.50E-03 | 3.50E-03 |  | 8.50E-03 |  |
| Zn | 3.00E-01 | 3.00E-01 | 3.00E-01 |  |  |  |
